# Defective IGF-1 prohormone N-glycosylation and reduced IGF-1 receptor signaling activation in congenital disorders of glycosylation

**DOI:** 10.1007/s00018-022-04180-x

**Published:** 2022-02-24

**Authors:** Laura Di Patria, Giosuè Annibalini, Amelia Morrone, Lorenzo Ferri, Roberta Saltarelli, Luca Galluzzi, Aurora Diotallevi, Matteo Bocconcelli, Maria Alice Donati, Rita Barone, Renzo Guerrini, Jaak Jaeken, Vilberto Stocchi, Elena Barbieri

**Affiliations:** 1 Department of Biomolecular Sciences, University of Urbino Carlo Bo, Via I Maggetti, 26/2, 61029 Urbino, Italy; 2 Laboratory of Molecular Biology of Neurometabolic Diseases, Neuroscience Department, Meyer Children’s Hospital, Florence, Italy; 3 Department of NEUROFARBA, University of Florence, Florence, Italy; 4 Child Neurology and Psychiatry Unit, Department of Clinical and Experimental Medicine, University of Catania, Catania, Italy; 5 Reseach Unit of Rare Diseases and Neurodevelopmental Disorders, Oasi Research Institute-IRCCS, Troina, Italy; 6 Center for Metabolic Diseases, University Hospital Gasthuisberg, KU Leuven, Leuven, Belgium; 7 Department of Human Sciences for the Promotion of Quality of Life, University San Raffaele, Roma, Italy; 8 IIM, Interuniversity Institute of Myology, Perugia, Italy

**Keywords:** IGF-1/IGF-1R signaling pathway, IGF-1 deficiency, N-linked glycosylation, Metabolic disorders, Rare genetic disease, Lectins

## Abstract

The insulin-like growth factor-1 (IGF-1) signaling pathway is crucial for the regulation of growth and development. The correct processing of the IGF-1Ea prohormone (proIGF-1Ea) and the IGF-1 receptor (IGF-1R) peptide precursor requires proper N-glycosylation. Deficiencies of N-linked glycosylation lead to a clinically heterogeneous group of inherited diseases called Congenital Disorders of Glycosylation (CDG). The impact of N-glycosylation defects on IGF-1/IGF-1R signaling components is largely unknown. In this study, using dermal fibroblasts from patients with different CDG [PMM2-CDG (*n* = 7); ALG3-CDG (*n* = 2); ALG8-CDG (*n* = 1); GMPPB-CDG (*n* = 1)], we analyzed the glycosylation pattern of the proIGF-1Ea, IGF-1 secretion efficiency and IGF-1R signaling activity. ALG3-CDG, ALG8-CDG, GMPPB-CDG and some PMM2-CDG fibroblasts showed hypoglycosylation of the proIGF-1Ea and lower IGF-1 secretion when compared with control (CTR). Lower IGF-1 serum concentration was observed in ALG3-CDG, ALG8-CDG and in some patients with PMM2-CDG, supporting our in vitro data. Furthermore, reduced IGF-1R expression level was observed in ALG3-CDG, ALG8-CDG and in some PMM2-CDG fibroblasts. IGF-1-induced IGF-1R activation was lower in most PMM2-CDG fibroblasts and was associated with decreased ERK1/2 phosphorylation as compared to CTR. In general, CDG fibroblasts showed a slight upregulation of Endoplasmic Reticulum (ER) stress genes compared with CTR, uncovering mild ER stress in CDG cells. ER-stress-related gene expression negatively correlated with fibroblasts IGF-1 secretion. This study provides new evidence of a direct link between N-glycosylation defects found in CDG and the impairment of IGF-1/IGF-1R signaling components. Further studies are warranted to determine the clinical consequences of reduced systemic IGF-1 availability and local activity in patients with CDG.

**Supplementary Information:**

The online version contains supplementary material available at 10.1007/s00018-022-04180-x.

## Introduction

The insulin-like growth factor-1 (IGF-1) signaling pathway is fundamental for growth regulation, especially in childhood, while also playing a central role in development and metabolic homeostasis. In the bloodstream, IGF-1 circulates primarily as part of a 150 kDa ternary complex with two additional proteins, the IGF binding protein-3 (IGFBP-3) and the acid-labile subunit (ALS). This system extends the half-life of circulating IGF-1 from 10 to 12 min to about 15 h, preserving stable serum levels of IGF-1 [1, 2]. Although growth hormone (GH) is a major regulator of hepatic IGF-1 production, IGF-1 is also regulated and secreted by other organs in an autocrine/paracrine manner [3]. Tissue-specific IGF-1 knockout models demonstrated the role of circulating IGF-1 in regulation of body size and tissue growth and revealed autocrine actions in specific tissues such as skeletal muscle [4], bone [5] and nervous system [6]. Thus, the activity of IGF-1 is due to a combination of local expression together with IGF-1 delivered to the tissue from the circulation where high levels are maintained [7].

The function of all the IGF-1 system components is affected by their glycosylation status [8, 9]. The mature IGF-1 is a non-glycosylated polypeptide of 7.6 kD derived from post-translational cleavage of the C terminal E peptide from the IGF-1 prohormone (proIGF-1E) [9]. In human, the most ubiquitously expressed prohormone, named proIGF-1Ea, is glycosylated (N-glycosylation site on residue 92 of the Ea petide) and proIGF-1Ea N-glycosylation ensures proper secretion of the mature IGF-1 [9, 10]. Both IGFBP-3 and ALS proteins are glycosylated. IGFBP-3 contains three N-glycosylation sites [1] and ALS is a soluble glycoprotein of 85 kDa [11]. Previous studies have demonstrated that N-glycosylation prolongs the half-life of IGFBP-3 [12], increases the affinity of ALS to the IGFBP-3/IGF-1 complex [13] and thus is required for the correct formation and stabilization of the 150 kDa ternary complex [2]. The actions of IGF-1 are regulated by the tyrosine kinase receptor IGF-1R. This receptor is synthesized as a single polypeptide precursor (proreceptor), which undergoes proteolytic cleavage into α (130 KDa) and β (97 kDa) chains that form a tetramer (α-β-β-α). Each α and β subunit contains 11 and 5N-glycosylation sites, respectively [14]; therefore, the α-β-β-α tetramer structure may enclose 32 glycosylation positions [15]. Several studies demonstrated that proper N-glycosylation of IGF-1R proreceptor is required for correct IGF-1R maturation and transport to the cell surface [15–17].

Defects in either IGF-1 or its receptor can result in poor pre- and post-natal growth [18, 19]. Beside growth failure, patients with IGF-1R haploinsufficiency often exhibit additional clinical features including skeletal malformation, intellectual disability, cardiac defects and facial dysmorphisms [19, 20]. In contrast, much less is known about the impact of N-glycosylation genetic defects, such as those found in Congenital Disorders of Glycosylation (CDG), on the IGF-1 system. CDG are a large family of rare inborn errors of metabolism caused by defective glycoprotein and glycolipid glycan synthesis and attachment. They comprise a broad spectrum of clinical manifestations, because glycosylation occurs in every cell and involves all organs and tissues [21]. Major clinical manifestations include nearly always neurological involvement (such as developmental/intellectual disability, hypotonia, epilepsy, stroke-like episodes, polyneuropathy) besides other organ involvement [21]. Nearly 160 CDG have been described and their number increases exponentially [22]. Glycosylation comprises a large number of biochemical pathways, including N-linked and O-linked pathways, glycosylphosphatidylinositol (GPI) anchor synthesis and glycolipid glycosylation. It occurs in several organelles: the cytoplasm, the endoplasmic reticulum (ER) and ER–Golgi intermediate compartment (ERGIC), the Golgi and vesicular network and the plasma and sarcolemmal membranes [23]. Most CDG are ultrarare disorders, those affecting N-glycosylation are the most common. Several specific therapeutic strategies are under evaluation for these diseases [24, 25]. PMM2-CDG is the most common N-glycosylation defect with more than 900 reported patients worldwide [21].

N-linked glycosylation disorders are usually identified by analyzing the serum transferrin isoforms by isoelectric focusing (IEF) or mass spectrometry [23]. On the basis of the transferrin IEF profiles (type 1 or type 2 pattern), N-glycosylation defects are classified as CDG-I or CDG-II. CDG-I results from defects in the glycan assembly in the cytoplasm and ER and is hallmarked by the absence of one or more N-glycans on glycoproteins [23]. CDG type-II is Golgi-related and characterized by defective glycans [23]. Hypoglycosylation leads to protein misfolding and a mild increase of ER-stress markers in patients’ fibroblasts [26].

Growing evidence suggests that insufficient levels and/or activity of growth factors might contribute to some of the clinical manifestations in CDG [8, 27–29]. The endocrine data from PMM2-affected children showed that circulating IGF-1 levels were in the low or low normal range in infants and children below the age of 9 years (*n* = 15 median, 33 µg/L; range, 12–92 µg/L; normal, 77–135 µg/L) and in the low-to-normal IGF-1 levels in adolescents (*n* = 8; median, 267 µg/L; range, 107–437 µg/L; normal, 350–504 µg/L) [29]. Similarly, Miller et al. [8] reported reduced serum levels of ALS, IGFBP-3, IGF-1 and of ternary complex formation in children with PMM2-CDG (*n* = 12; age = 8.1 ± 6.6 years) compared with age-matched controls, despite normal or elevated levels of GH. These features were also found in the recently developed mouse model of PMM2-CDG [28].

The molecular basis of the IGF-1 system deficiency found in PMM2-CDG patients is currently unknown.

In this study, we investigated whether glycosylation is directly involved in the function of the IGF-1 system. We hypothesize that primary fibroblasts from patients with CDG show aberrant proIGF-1Ea N-glycosylation and impaired activation of the IGF-1R signaling pathways.

## Materials and methods

### CDG fibroblasts

Primary fibroblasts derived from patients with CDG caused by different N-glycosylation defects were studied. ALG3-CDG (α-1,3-mannosyltransferase deficiency), PGAP2-CDG (post-GPI attachment to proteins 2 deficiency), ALG8-CDG (α-1,3-glucosyltransferase 2 deficiency), GMPPB-CDG (GDP-mannose pyrophosphorylase B) and control (CTR) fibroblasts from age-matched volunteers were provided by the Neuroscience Department of the Meyer Children’s Hospital (Florence, Italy). PMM2-CDG (phosphomannomutase-2 deficiency) fibroblasts were obtained from the Giannina Gaslini Institute-Telethon Network of Genetic Biobanks (Genoa, Italy) [30]. Clinical features of these patients have been previously published in part [31] and are described in the Supplementary Table 1. Fibroblasts were cultured in Dulbecco’s Modified Eagle Medium (DMEM, high glucose) supplemented with 10% heat-inactivated fetal bovine serum (FBS), 2 mM glutamine, penicillin (100 U/mL) and streptomycin (100 μg/mL) and maintained in a 5% CO_2_ atmosphere at 37 °C. For IGF-1R activation, after 24 h of serum-free culture in 12-well plates, cells were stimulated with IGF-1 (100 ng/mL) (Cat #I3769-50UG, Sigma-Aldrich) for 30 or 60 min before lysis.

For IGF-1 gene overexpression, fibroblasts were transfected using the Human Dermal Fibroblast Nucleofector™ Kit (Cat #VPD1001; Lonza) using the Nucleofector™ Device (Lonza) following the instruction manual. Briefly, 1 × 10^6^ fibroblasts were resuspended in the nucleofector solution, combined with 2.5 µg of plasmid construct containing the class 1 IGF-1Ea isoform [32], transferred to the cuvette supplied and finally transfected with the U-23 program of the Nucleofector™ Device. After electroporation, cells were mixed with 500 µl of DMEM and immediately plated into a 6-well plate. The efficiency of transfection was estimated by GFP-dependent fluorescence and by qPCR for total IGF-1 mRNA levels at 48 h after transfection, as previously described [32]. The fibroblasts' concentration and cell viability were determined by the LUNA-II™ Automated Cell Counter (Logos Biosystems, Twin Helix) with trypan blue staining.

### ELISA assay

Quantitative determination of IGF-1 concentrations in transfected fibroblasts supernatants was performed by a commercially available ELISA kit following the manufacturer’s instructions (Human IGF-I/IGF-1 DuoSet ELISA Cat #DY291-05; R&D Systems). The data were acquired at a wavelength of 450 nm using Model 680 microplate reader (Bio-Rad Laboratories).

### Western blot and lectin blot analyses

Fibroblasts were processed for western blot analyses as previously reported [32]. The protein samples (20–40 µg total proteins) were electrophoresed with 10% SDS-PAGE and then transferred to nitrocellulose or PVDF membranes (Bio-Rad Laboratories) for immunoblotting. Primary antibodies against phospho-IGF-1 Receptor β (1:2000; Cat #3024 Cell Signaling Technology); IGF-1 Receptor β (1:2000; Cat #3027 Cell Signaling Technology), phospho-Akt (Ser473) (1:2000; Cat #9271 Cell Signaling Technology), Akt (1:2000; Cat #9272 Cell Signaling Technology), phospho-p44/42 (ERK1/2) (1:2000; Cat #9101 Cell Signaling Technology), p44/42 (ERK1/2) (1:2000; Cat #9102 Cell Signaling Technology) and IGF-1 (1:2000; Cat #500P11 Peprotech) were incubated overnight at 4 °C. For lectin blotting, membranes were probed with biotinylated Concanavalin A (ConA, 1:1000; Cat #B-1005-5) and *Phaseolus vulgaris* leucoagglutinin (PHA-L, 1:200; Cat #B-1115-2) lectins (Vector laboratories, D.B.A. Italia) at room temperature while shaking for 1 h. After washes, the membranes were incubated with the appropriate horseradish peroxidase (HRP)-conjugated secondary antibody (against primary antibodies) or streptavidin–HRP (for biotinylated lectins) at room temperature for 1 h and were then washed three times. Blots were developed using Clarity Western ECL Substrate (Bio-Rad Laboratories) and were quantified using the ChemiDoc MP (Bio-Rad Laboratories) equipped with Image Lab software*.*

### RNA extraction, cDNA synthesis and quantitative real-time polymerase chain reaction

The total RNA was extracted and genes of interest analyzed by real-time qPCR as previously described [33]. Briefly, the expression of ER-stress-related genes was monitored by qPCR using TB Green Premix Ex TaqII Mastermix (Takara Bio Europe, France), in a RotorGene 6000 instrument (Corbett life science, Sydney, Australia). As template, 2 ng of total RNA used for cDNA synthesis was used in each PCR tube and a non-template control was included for each primer pair reaction as negative control. All amplification reactions were performed in duplicate. The amplification conditions were: 95 °C for 10 min, 95 °C for 10 s and 60 °C for 50 s for 40 cycles. At the end of each run, a melting curve analysis from 65 °C to 95 °C was performed to exclude the presence of non-specific products or primer dimers. The data were normalized against the reference gene GAPDH (glyceraldehyde-3-phosphate dehydrogenase). The relative expression levels were calculated using the 2^−ΔΔCt^ method [34].

### Statistical analyses

The data are represented as mean ± SEM of at least three independent experiments. Statistical analyses were performed using the *t* test or one-way ANOVA as appropriate, followed by Tukey’s Multiple Comparison post hoc test. Correlation between fibroblasts IGF-1 secretion, densitometric quantification of proIGF-1Ea and IGF-1R immunoreactive bands, lectin binding and ER-stress-related genes was analyzed using Pearson’s correlation. The statistical tests were performed using SPSS (IBM SPSS Statistics for Windows, Version 20.0, IBM Corp.) and GraphPad Prism version 5 (GraphPad Software, Inc., La Jolla, CA, USA). A *p* value ≤ 0.05 was considered statistically significant.

## Results

### proIGF-1Ea N-glycosylation pattern and mature IGF-1 secretion in CDG fibroblasts

To evaluate the expression level of IGF-1 in CTR and CDG fibroblasts, we conducted preliminary analyses on the IGF-1 mRNA and protein quantity. IGF-1 mRNA quantification by qPCR showed that all fibroblasts had detectable levels of IGF-1 mRNA (mean CT value 30.5); however, the IGF-1 protein level was too low to be detected by western blot or ELISA. We also tried to quantify the IGF-1 secretion in the fibroblast supernatants after concentration using 3 kDa Spin Columns (by about 20-fold; Amicon^®^ Ultra Merck Millipore), but the IGF-1 concentration still remained below the ELISA sensitivity (93.8 pg/mL). Subsequently, to assess the expression pattern and the N-glycosylation status of the IGF-1 protein, we transiently transfected fibroblasts with a plasmid encoding the IGF-1Ea isoform or an empty vector [32] by electroporation.

**Fig. 1:**
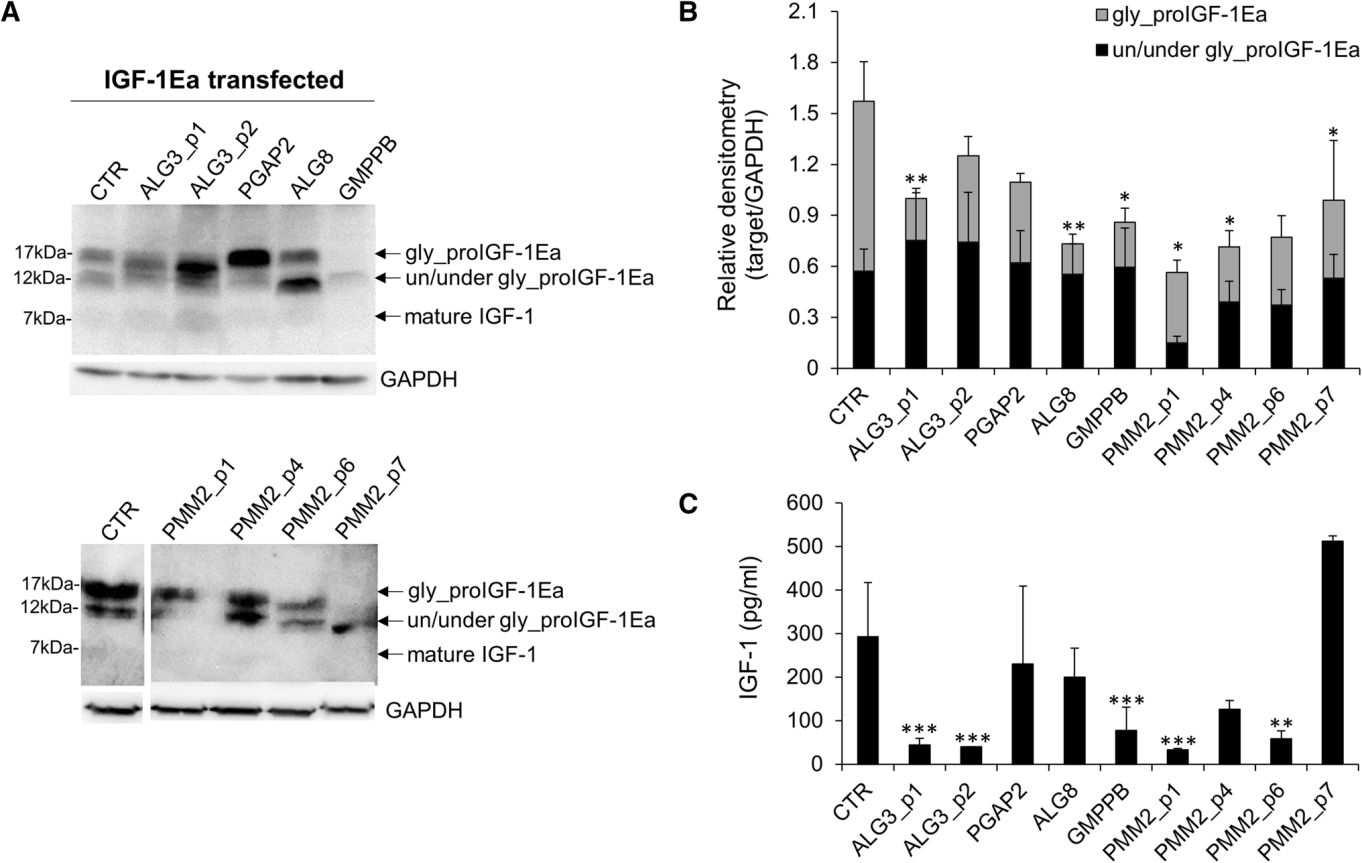
IGF-1Ea prohormone expression patterns and IGF-1 secretion. Representative western blot showing the IGF-1Ea prohormone expression patterns (**A**) and relative protein expression level (**B**) of IGF-1Ea transfected fibroblasts (mean ± SEM of three technical replicates for each fibroblast subtype). Quantification of IGF-1 level in fibroblast supernatants measured by ELISA (C) (mean ± SEM of three technical replicates for each fibroblast subtype). Gly_proIGF-1Ea: glycosylated IGF-1Ea prohormone; un/under gly_proIGF-1Ea: un/underglycosylated IGF-1Ea prohormone. *significantly different from CTR fibroblasts; ***p < 0.001, **p < 0.01, *p < 0.05

As shown in Fig. 1A, two distinct bands, likely representing glycosylated (~ 17 kDa) and unglycosylated (~ 12 kDa) proIGF-1Ea, were detected in CTR and PGAP2-CDG fibroblasts. In contrast, the glycosylation pattern of proIGF-1Ea differed in other CDG fibroblasts. In particular, the two fibroblasts from ALG3-CDG presented a distinct band of about 14 kDa, while ALG8-CDG and GMPPB-CDG revealed a marked accumulation of 12 kDa proteins. It is likely that all these bands represent underglycosylated forms of the proIGF-1Ea. Among PMM2-CDG fibroblasts analyzed, there was a general preservation of proIGF-1Ea N-glycosylation pattern, however the IGF-1 bands intensity in PMM2_p1-CDG, PMM2_p6-CDG and PMM2_p7-CDG fibroblasts were lower compared to CTR. Densitometric analysis of western blot bands further confirmed a reduction of glycosylated proIGF-1Ea band in most CDG fibroblasts compared to CTR (Fig. 1B), while the intensities of un/under-glycosylated bands did not change (ANOVA, *p* = 0.101). Subsequently, we quantified the IGF-1 protein level in the cell culture media of IGF-1Ea-transfected fibroblasts (Fig. 1C). We observed that both ALG3-CDG fibroblasts derived from two different patients, GMPPB-CDG, PMM2_p1-CDG and PMM2_p6-CDG showed a decreased IGF-1 secretion as compared to CTR. The secretion pattern of IGF-1 positively correlated with the glycosylated proIGF-1Ea quantity (*r* = 0.612, *p* = 0.020), corroborating the previous finding that N-glycosylation is required for proper proIGF-1Ea processing and IGF-1 secretion [9, 10]. IGF-1 serum level was also quantified from some available patients (Supplementary Table 1). Low serum IGF-1 was found in ALG3-CDG (52 ng/mL, normal 95–312 ng/mL) and ALG8-CDG patients (80 ng/mL, normal, 95–460 ng/mL), while normal levels were observed in PGAP2-CDG (116 ng/mL, normal 47–231 ng/mL). Among PMM2-CDG patients, decreased IGF-1serum levels were observed in patients 2 (64 ng/ml, normal 99–238 ng/ml) and 4 (80 ng/ml, normal 99–238 ng/ml) while it was in the low normal or normal range in patients 1 (101 ng/ml, normal 82–214 ng/ml) and 7 (361 ng/mL, normal 119–395 ng/mL), respectively.

### IGF-1R expression and IGF-1R pathway activation in CDG fibroblasts

**Fig. 2:**
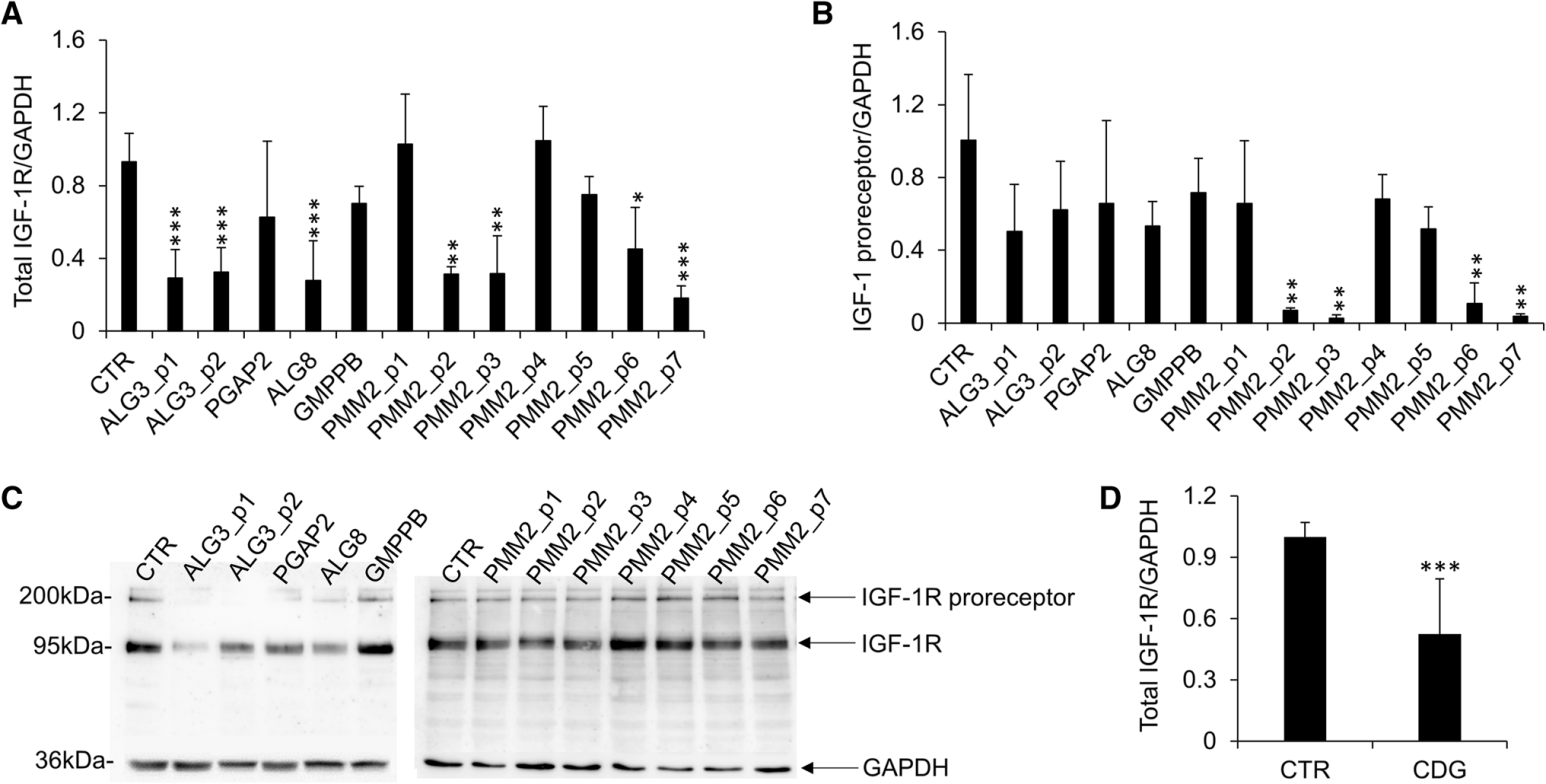
IGF-1R protein levels in CDG fibroblasts. Relative expression level of IGF-1R (**A**) and IGF-1R proreceptor (**B**) in different CDG quantified by western blot (mean ± SEM of three technical replicates for each fibroblast subtype). Representative western blot showing expression levels of IGF-1R proreceptor (~ 200 kDa), IGF-1R (~ 97 kDa) and GAPDH (~ 36 kDa) (**C**). Relative expression levels of IGF-1R in CTR (mean of three technical replicates for each of five biological replicates) and CDG- fibroblasts (mean of three technical replicates for each of twelve biological replicates) quantified by western blot (**D**). *significantly different from CTR fibroblasts; ***p < 0.0001, **p < 0.001, *p < 0.05

**Fig. 3:**
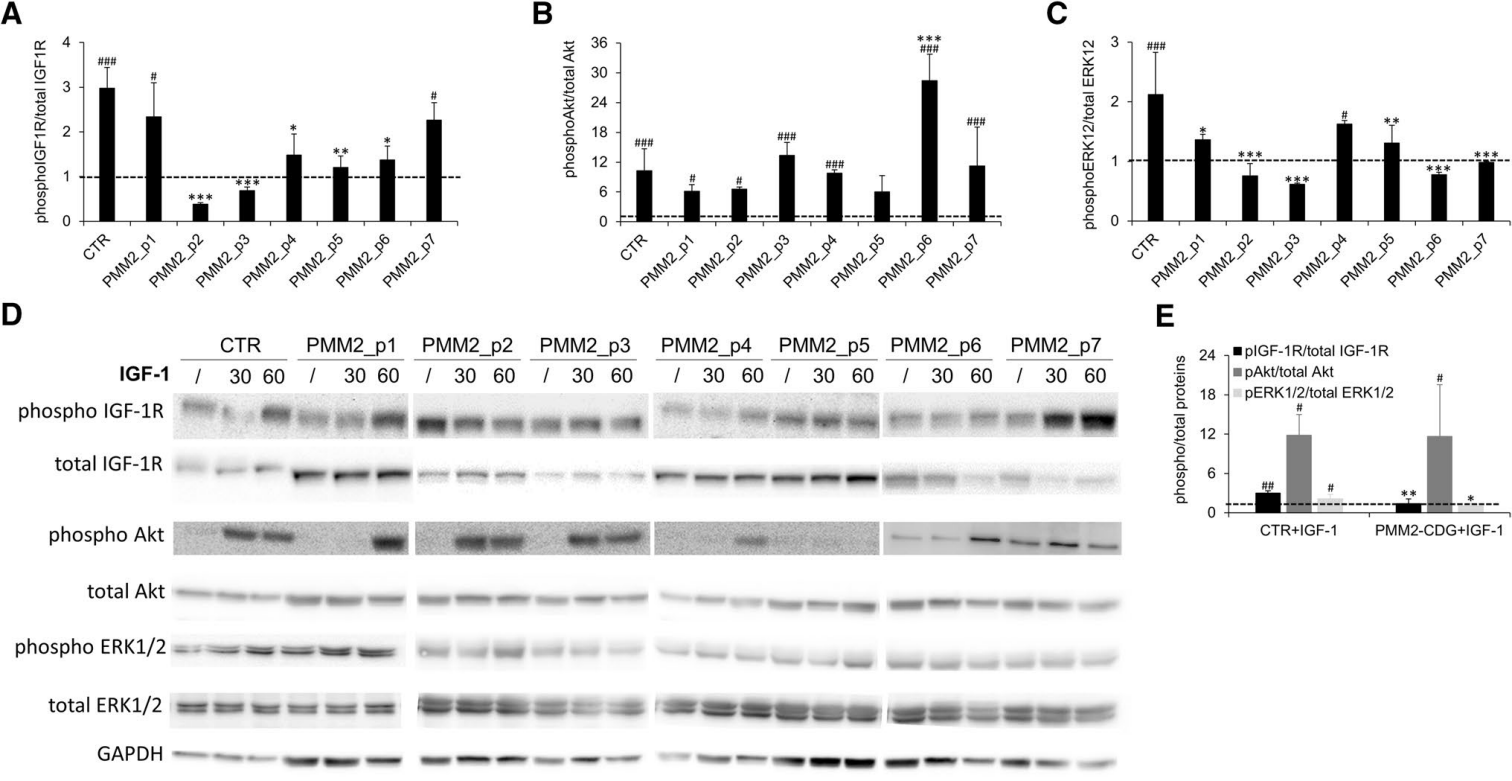
IGF-1R signaling pathway activation. Relative expression levels of phosphorylated IGF-1R (**A**), Akt (**B**) and ERK1/2 (**C**) in PMM2-CDG fibroblasts obtained from different patients (mean ± SEM of three technical replicates for each fibroblast subtype). Representative western blot showing expression levels of phosphorylated and total IGF-1R, Akt, ERK1/2 and GAPDH (**D**). IGF-1R, Akt and ERK1/2 phosphorylation level in CTR (mean of three technical replicates for each of five biological replicates) and PMM2-CDG (mean of three technical replicates for each of seven biological replicates) fibroblasts (**E**). *significantly different from CTR fibroblasts; #significantly different from recombinant IGF-1 untreated cells *** and ^###^*p* < 0.0001, ^##^**p < 0.001, *p < 0.01; ^#^*p* < 0.05

**Fig. 4:**
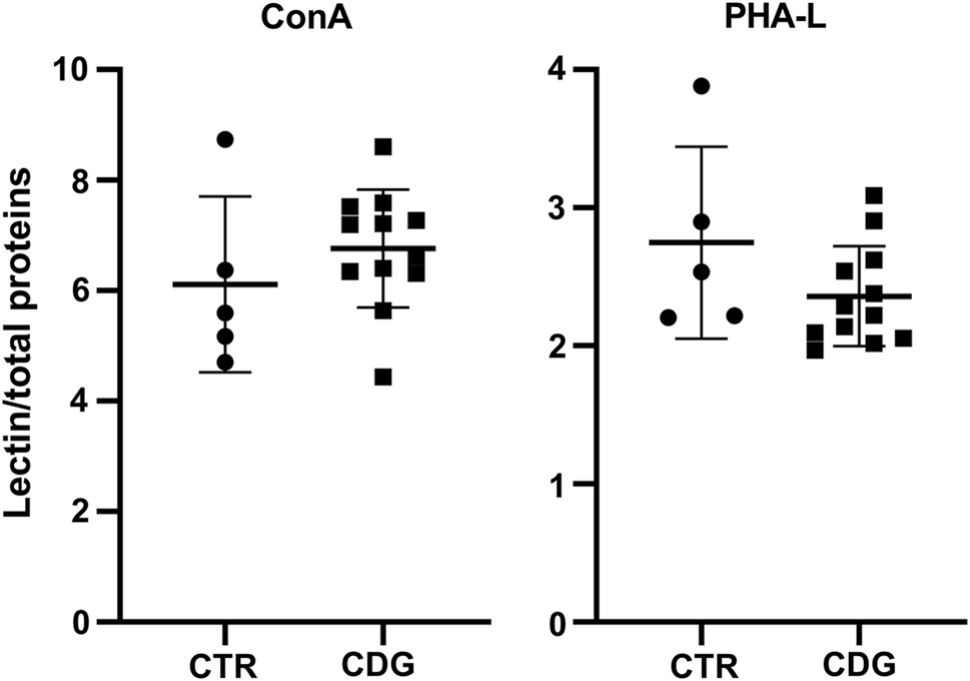
Lectin-binding analysis. Concanavalin A (ConA) and Phaseolus vulgaris leucoagglutinin (PHA-L) binding to CTR (mean of three technical replicates for each of five biological replicates) and CDG (mean of three technical replicates for each of twelve biological replicates) fibroblasts. Con A and PHA-L recognize high mannose and complex type N-glycans, respectively. *significantly different from CTR fibroblasts, *p < 0.0001

As shown in Fig. 2A, ALG3-CDG fibroblasts from two different patients and ALG8-CDG cells revealed reduced IGF-1R expression levels compared to CTR fibroblasts. PMM2-CDG fibroblasts from different patients presented significant variability in the IGF-1R expression levels. IGF-1R was reduced in about 50% of PMM2-CDG cells tested. Reduced levels of IGF-1R found in CDG fibroblasts were not followed by an IGF-1R proreceptor accumulation (Fig. 2B and C). Altogether, CDG fibroblasts showed reduced IGF-1R expression compared with CTR (Fig. 2D). To further investigate the possible differences in the two main study CDG subtypes, we performed a post hoc subgroup analysis for ALG-CDG (*n* = 3) and PMM2-CDG (*n* = 7) showing reduced IGF-1R levels both in ALG-CDG (*p* = 0.003) and PMM2-CDG (*p* = 0.02). To evaluate if IGF-1R deficiency observed in PMM2-CDG was associated with reduced IGF-1R pathway activation, fibroblasts were treated with recombinant IGF-1 (100 ng/ml) for 30 and 60 min and the level of IGF-1R (Fig. 3A), Akt (Fig. 3B) and ERK1/2 (Fig. 3C) phosphorylation was quantified by western blotting. The IGF-1-induced activation of IGF-1R and ERK1/2 was reduced in most PMM2-CDG fibroblasts, while Akt phosphorylation level was more heterogeneous. Notably, in some PMM2-CDG, the decreased response to IGF-1 treatment was mainly due to higher basal IGF-1R and ERK1/2 activation (e.g., PMM2_p2-CDG; PMM2_p3-CDG; PMM2_p5-CDG; PMM2_p6-CDG), instead of general inhibition of IGF-1R and ERK1/2 activity (Fig. 3D). When PMM2-CDG fibroblasts derived from different patients were pooled together (*n* = 7), both IGF-1R and ERK1/2 IGF-1-induced activation were reduced compared to CTR (Fig. 3E). Preliminary results obtained in some ALG-CDG fibroblasts showed IGF-1 pathway downregulation (not shown) (Fig. 4).

### Lectin binding analysis and ER-stress-related markers in CDG fibroblasts

**Fig. 5:**
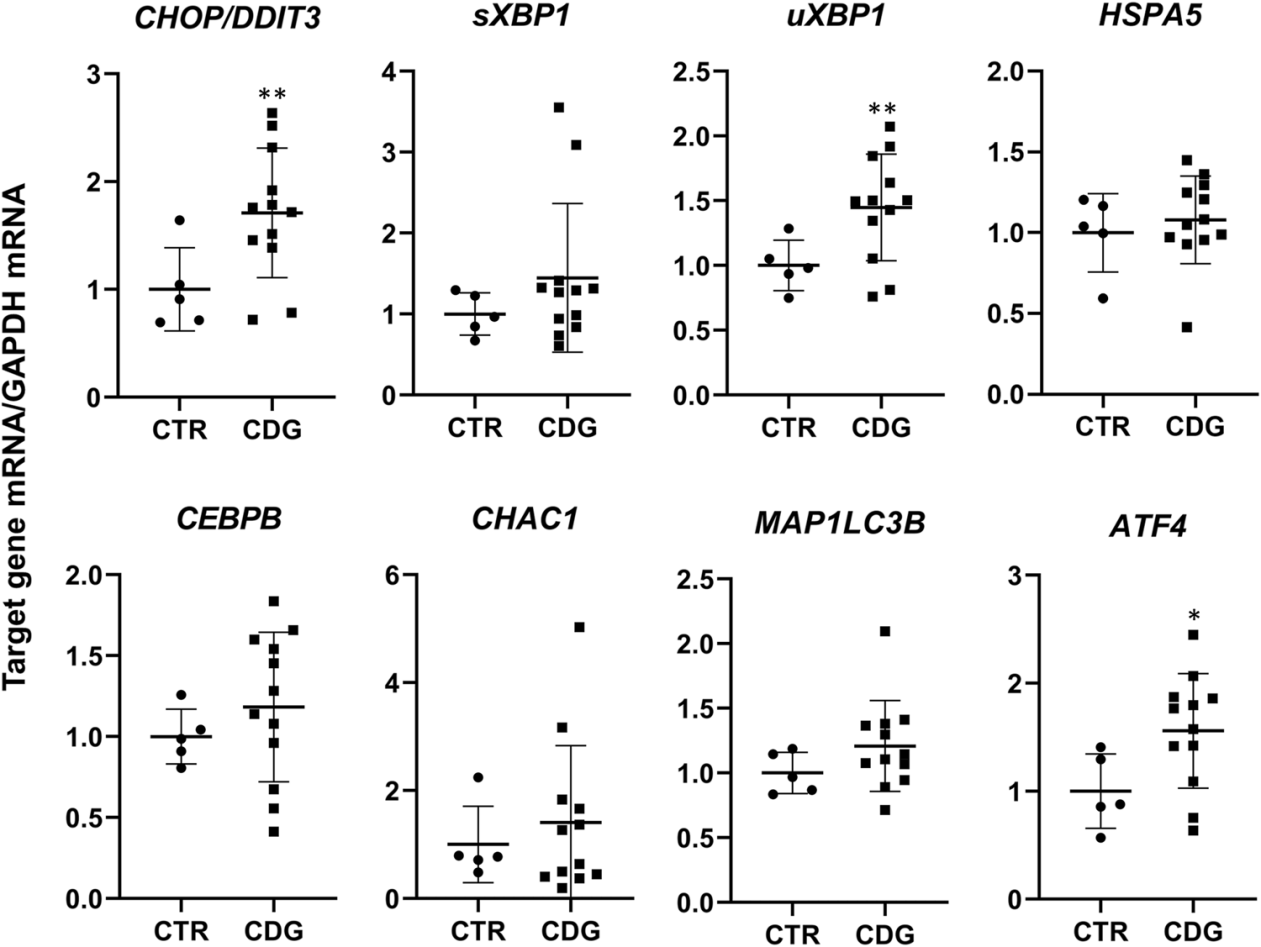
ER-stress markers' mRNA expression in CDG fibroblasts. Quantification of the expression level of selected ER-stress-related genes: *CHOP/DDIT3*, *sXBP1*, *uXBP1*, *HSPA5, CEBPB, CHAC1, MAP1LC3B* and *ATF4* in CTR (mean of three technical replicates for each of five biological replicates) and CDG (mean of three technical replicates for each of 12 biological replicates) fibroblasts. *significantly different from CTR fibroblasts, **p < 0.01, *p < 0.05

Lectin blotting with ConA and PHA-L, which recognize high mannose and beta 1,6 branched oligosaccharides complex type N-glycans, respectively, showed a non-significant decrement trend of PHA-L binding in CDG- compared to CTR- fibroblasts (*p* = 0.29), while ConA reactivity did not change (*p* = 0.43). Subsequently, qPCR was carried out to quantify the expression level of selected ER-stress-related genes: *CHOP/DDIT3*, *sXBP1*, *uXBP1*, *MAP1LC3B*, *HSPA5*, *CEBPB*, *CHAC1* and *ATF4*. As shown in Fig. 5, *CHOP/DDIT3, uXBP1* and *ATF4* mRNA levels were higher in CDG compared to CTR fibroblasts. The post hoc subgroup analysis showed that *ATF4* mRNA expression was higher in ALG-CDG as compared to PMM2-CDG and CTR fibroblasts (*p* = 0.008), while *CHOP/DDIT3 and uXBP1* mRNA levels did not differ between ALG-CDG and PMM2-CDG. The Pearson’s correlation coefficient showed that fibroblast IGF-1 secretion was directly associated with PHA-L binding (*r* = 0.516, *p* = 0.05) and inversely correlated with *CHOP/DDIT3* (*r* = − 0.747, *p* = 0.002), *uXBP1* (*r* = − 0.715, *p* = 0.004), *CEBPB* (*r* = − 0.548, *p* = 0.043) and *ATF4* (*r* = − 0.735, *p* = 0.003) mRNA expression.

## Discussion

In this study, we provide new evidence for IGF-1 system impairment in CDG fibroblasts due to defective N-glycosylation of proIGF-1Ea and IGF-1R, which partially disrupts IGF-1 signaling pathway activation. We previously demonstrated that, under physiological conditions, intracellular IGF-1 is mainly expressed as a heavily N-glycosylated prohormone of 17–22 kDa [10]. Here, using CTR and CDG fibroblasts transiently transfected with the IGF-1Ea isoform, we monitored the N-glycosylation pattern of proIGF-1Ea and IGF-1 secretion. Two distinct bands, likely representing glycosylated and unglycosylated proIGF-1Ea, were found in CTR, PGAP2-CDG and most PMM2-CDG fibroblasts by western blotting. Conversely, a unique band was present in fibroblasts derived from ALG3-CDG, ALG8-CDG and GMPPB-CDG. In both ALG3-CDG, we found an abnormal band of about 14 kDa, while in ALG8-CDG and in GMPPB-CDG, we found a marked accumulation of a 12 KDa isoform. These bands probably represent hypoglycosylated isoforms of the proIGF-1Ea. To evaluate if aberrant proIGF-1Ea glycosylation impaired IGF-1 secretion, we quantified the IGF-1 protein level in cell culture supernatants and found that most CDG fibroblasts released less IGF-1 in the culture media compared to CTR. These data corroborate our previous findings that N-glycosylation of the proIGF-1Ea is essential for correct IGF-1 secretion [10]. Normal proIGF-1Ea N-glycosylation and IGF-1 secretion in PGAP2-CDG fibroblasts was expected, since PGAP2 is not involved in N-glycosylation but in GPI-anchor synthesis of GPI-anchored proteins. The IGF-1 serum level was evaluated in seven patients, whose fibroblasts have been included in this work (ALG3-CDG, ALG8-CDG, PGAP2-CDG and PMM2_p1,_p2,_p4,_p7-CDG). ALG3-CDG and ALG8-CDG patients showed low IGF-1 serum levels, while low-to-normal IGF-1 serum levels were found in studied patients with PMM2-CDG. These data remain provisional, due to the low number of patients tested, but they are in line with the published data on reduced circulating IGF-1 levels in CDG patients [8, 29].

Because the effects of IGF-1 are mediated principally through the IGF-1R, we subsequently analyzed the IGF-1R levels in primary fibroblasts from different CDG. Previous studies have shown that N-glycosylation contributes to ligand binding, kinase activity and turnover of the IGF-1R [35]. Accordingly, we found that IGF-1R expression and activity were reduced in primary fibroblasts from different CDG. Levels of IGF-1R were particularly low in ALG3-CDG, ALG-8-CDG and in some of the PMM2-CDG fibroblast cell lines tested. We did not find an accumulation of the IGF-1 proreceptor in these cells, suggesting that defective N-glycosylation causes IGF-1R proreceptor instability and degradation [27]. In PMM2-CDG, we also analyzed the IGF-1-induced IGF-1R pathway activation. Most PMM2-CDG fibroblasts showed reduced IGF-1R and ERK1/2 phosphorylation compared to CTR, while the Akt activation did not differ significantly probably due to the high variability of the Akt response among PMM2-CDG fibroblasts.

Altogether, these data corroborate and expand previous observations demonstrating an IGF-1 system deficiency in CDG [8, 27–29]. In particular, our results suggest that the glycosylation defect found in CDG might impair the IGF-1 system not only indirectly, by destabilizing the ternary complex [8], but also directly due to proIGF-1Ea hypoglycosylation and IGF-1 secretory defect. Furthermore, local growth-promoting effects of IGF-1 might be compromised by the reduced IGF-1R proreceptor glycosylation and related impairment of IGF-1 signal transduction. We believe that these findings are relevant in the context of CDG since both systemic IGF-1 production and local bioactivity are essential to support normal growth during development [5, 7]. Patients with genetic IGF-1R defects, show symptoms (i.e., pre and postnatal growth retardation, microcephaly, cardiac defects and dysmorphic features) that significantly overlap with those presented in various CDG types [19, 20]. For example, the majority of CDG patients analyzed in the present study showed growth failure and microcephaly (Supplementary Table 1), which are also clinical hallmarks of IGF-1/IGF-1R defects [36–38]. Further studies are needed, also taking advantage of the recently developed CDG animal models [28], to evaluate the impact of glycosylation defects on each component of the IGF-1 system. This, in turn, will help to clarify the association between clinical CDG features and IGF-1/IGF-1R signaling abnormalities. Interestingly, a case study showed that treatment with recombinant IGF-1 prompted linear growth in a child with PMM2-CDG [39], suggesting the potential of IGF-1 therapy in the improvement of clinical outcome of CDG patients. However, taking into account our data together with previous findings [27], the residual IGF-1R activity should be measured before starting a recombinant IGF-1 therapy.

Although typical clinical features of CDG were present in patients whose fibroblasts we studied, clinical manifestations and severity have a wide spectrum, which is probably related to the specific gene variants and/or enzyme residual activity [30 and Supplementary Table 1]. Global quantitative protein glycosylation performed with lectins that have specific affinity for different types of N-glycans (ConA and PHA-L) failed to highlight difference between different CDG fibroblast subtypes. We found a modest, and not significant, decrease of PHA-L reactivity, indicating reduced levels of β1–6 branching structures and complex N-glycans. A positive association between IGF-1 secretion and PHA-L binding was found. In addition, the analysis of ER-stress-related gene expression showed a slight increase of *CHOP/DDIT3*, *uXBP1* and *ATF4* mRNA level in CDG compared to CTR fibroblasts, which negatively correlate with fibroblasts IGF-1 secretion. A correlation between ER stress and IGF-1 system alterations has been proposed in several neurodegenerative diseases that share as common feature the accumulation of misfolded proteins [40]. Although further studies are needed, the broad modification of protein N-glycosylation patterns and the mild ER-stress in CDG fibroblasts might also have contributed to the IGF-1/IGF-1R impaired signaling observed in CDG fibroblasts [40, 41].

## Conclusions

In this study, we have shown that primary fibroblasts from CDG patients have reduced levels of proIGF-1Ea and IGF-1R proreceptor glycosylation. The decreased glycosylation of IGF-1/IGF-1R signaling pathway components reduced the IGF-1 bioactivity in CDG fibroblasts. Thus, our molecular data provide new insight into CDG pathogenesis and suggest that both IGF-1 production (circulating/local) and bioactivity might be compromised in CDG due to reduced N-glycosylation.

These findings pave the way to future studies focused on the impact of CDG on the different IGF-1 system components, which include IGF-1, IGFBPs and the IGF-1R. More efforts will be needed to clarify the correlation between reduced IGF-1/IGF-1R signaling in CDG patients and their clinical manifestations.

## Supplementary Information

Below is the link to the electronic supplementary material.

Supplementary file1 (DOCX 22 KB)

## Data Availability

The datasets generated during and/or analyzed during the current study are available from the corresponding author on reasonable request.

## Abbreviations

Akt: RAC-alpha serine/threonine-protein kinase
ALG3: Dol-P-Man:Man(5)GlcNAc(2)-PP-Dol alpha-1,3-mannosyltransferase
ALG8: Dolichyl pyrophosphate Glc1Man9GlcNAc2 alpha-1,3-glucosyltransferase
ALS: Acid-labile subunit
ATF4: Activating transcription factor 4
CDG: Congenital Disorder(s) of Glycosylation
CEBPB: CCAAT enhancer binding protein beta
CHAC1: ChaC glutathione-specific gamma-glutamylcyclotransferase 1
CHOP/DDIT3: DNA damage inducible transcript 3
ConA: Concanavalin A
CTR: Control
DMEM: Dulbecco’s Modified Eagle Medium
ER: Endoplasmic reticulum
ERGIC: ER–Golgi intermediate compartment
ERK: Mitogen-activated protein kinase
FBS: Fetal bovine serum
GAPDH: Glyceraldehyde-3-phosphate dehydrogenase
GH: Growth hormone
GMPPB: GDP-mannose pyrophosphorylase
GPI: Glycosylphosphatidylinositol
HRP: Horseradish peroxidase
HSPA5: Heat shock protein family A (Hsp70) member 5
IEF: Isoelectric focusing
IGF-1: Insulin-like growth factor-1
IGF1-R: Insulin-like growth factor-1 receptor
IGFBP-3: IGF binding protein-3
MAP1LC3B: Microtubule associated protein 1 light chain 3 beta
PGAP2: Post-GPI attachment to proteins factor 2
PHA-L: *Phaseolus vulgaris* Leucoagglutinin
PMM2: Phosphomannomutase 2
proIGF-1E: IGF-1 prohormone
sXBP1: Spliced X-box binding protein 1
uXBP1: Unspliced X-box binding protein 1
