# Supplementary material for: Defective IGF-1 prohormone N-glycosylation and reduced IGF-1 receptor signaling activation in congenital disorders of glycosylation
Source: Cell Mol Life Sci. 2022 Feb 24;79(3):150. doi: 10.1007/s00018-022-04180-x (PMC8873121; doi:10.1007/s00018-022-04180-x)
Supplement: Supplementary file 1 — Supplementary file1 (DOCX 22 KB) [file 18_2022_4180_MOESM1_ESM.docx]

**Supplementary table 1: Clinical features of CDG patients analysed in the present study**

|  | **Patient code/sex** | **Age at last examination** | **Skin fibroblast isolation** | **Clinical findings** | **Growth failure** | **Weight (kg)/Height (cm) at last examination**  **[SDs from average]** | **GH (ng/mL)**  **[normal range]** | **IGF-1 (ng/mL)**  **[normal range]** | **IGFBP-3 (µg/mL)**  **[normal range]** | **Gene variants** | **Diagnosis**  **(OMIM)** |
| --- | --- | --- | --- | --- | --- | --- | --- | --- | --- | --- | --- |
| 1 | ALG3_p1/M | 9 years | 2011 | Spastic quadriparesis, severe developmental delay, acquired microcephaly, (<4SD) | yes | 19/NA  [-3.8/NA] | 8.61  [0.09–1.95] | 52  [95-312] | 2.09  [1.89–7.33] | Homozygous *ALG3* c.1A>G p.(Met1?) | ALG3-CDG  (*608750) |
| 2 | ALG3_p2/F | Died at 6 years of age for respiratory failure during pneumonia | 2009 | Spastic quadriparesis, severe cognitive impairment, acquired microcephaly, (<4SD), facial dysmorphism, severe scoliosis, distal arthrogryposis, hepatomegaly | yes | NA | NA | NA | NA | Compound heterozygous *ALG3* c.101G>A  p.(Arg35His) / c.165C>T  p.(Gly55Gly) | ALG3-CDG  (*608750) |
| 3 | PGAP2/M | 5 years | 2014 | Severe hypotonia, epileptic encephalopathy, psychomotor delay, hyperphosphatasia | no | 19/107  [0.2/-0.5] | 1.41  [0.09–2.50] | 116  [47-231] | 3.16  [1.20–5.40] | Compound heterozygous *PGAP2* c.549dupA  p.(Gln184Thrfs*26) / c.686 C>T p.(Ala229Val) | PGAP2-CDG  (*615187) |
| 4 | ALG8/M | 8 years | 2014 | Mild hypotonia, facial dysmorphism, psychomotor delay, autism | no | 42/135  [2.3/1.3] | 0.07  [0.12–8.90] | 80  [95-460] | 4.45  [2.41–8.54] | Compound heterozygous *ALG8* c.122G>A  p.(Arg41Gln) / [c.446T>G  p.(Leu149Arg) + c.980C>G p.(Thr327Arg)] | ALG8-CDG |
| 5 | GMPPB/M | Died at 13 months for respiratory failure during pneumonia | 2016 | Severe hypotonia, congenital muscular dystrophy, facial dysmorphism, epileptic encephalopathy, distal arthrogryposis | yes | 5/59  [-5.3/-7.2] | NA | NA | N NA | Compound heterozygous *GMPPB* c.95C>T  p.(Pro32Leu)/ c.931C>T p.(Arg311Cys) | GMPPB-CDG  (*615320) |
| 6 | PMM2_p1_20706/M $# | 44 years | 1984 | Dysmorphic features, microcephaly, cerebellar atrophy, peripheral neuropathy, severe intellectual disability | yes | 58/152  [-1.2/-3.4] | NA | 101  [82-214] | NA | Val129Met/Arg141His | PMM2-CDG  (*601785) |
| 7 | PMM2_p2_20707/M $# | 37 years | 1984 | Dysmorphic features, microcephaly, cerebellar atrophy, peripheral neuropathy, severe intellectual disability | yes | 46/146  [-2.7/-4.3] | NA | 64  [99-238] | NA | Val129Met/Arg141His | PMM2-CDG  (*601785) |
| 8 | PMM2_p3_21197/M | NA | 1991 | NA | NA |  | NA | NA | NA | NA | PMM2-CDG  (*601785) |
| 9 | PMM2_p4_21213/M $ | 29 years | 1991 | Dysmorphic features, microcephaly, cerebellar atrophy, peripheral neuropathy, severe intellectual disability | yes | 46/147  [-2.7/-4.1] | NA | 80  [99-238] | NA | Val231Met/Arg141His | PMM2-CDG  (*601785) |
| 10 | PMM2_p5_22433/M |  | 2002 | NA | NA |  | NA | NA | NA | NA | PMM2-CDG  (*601785) |
| 11 | PMM2_p6_22540/F |  | 2003 | NA | NA |  | NA | NA | NA | NA | PMM2-CDG  (*601785) |
| 12 | PMM2_p7_23167/M $ | 17 years | 2004 | Dysmorphic features, microcephaly, cerebellar atrophy, peripheral neuropathy, moderate intellectual disability | yes | 32/140  [-4.1/-4.8] | NA | 361  [119-395] | NA | Arg141His/Asn216Ile | PMM2-CDG  (*601785) |

$ Reported in Barone et al. J Neurol 2015 (Table 1 pts. 4,5,7, and 3 respectively). M male; F female; # Sibling pairs; NA not assessed;. SDs standard deviation scores from average.
